# Hospitalizations in patients with idiopathic pulmonary fibrosis

**DOI:** 10.1186/s12931-021-01851-4

**Published:** 2021-09-30

**Authors:** Hyun J. Kim, Laurie D. Snyder, Ayodeji Adegunsoye, Megan L. Neely, Shaun Bender, Eric S. White, Craig S. Conoscenti, Mary E. Strek, The IPF-PRO Registry Investigators, Albert Baker, Scott Beegle, John A. Belperio, Rany Condos, Francis Cordova, Daniel A. Culver, Daniel Dilling, John Fitzgerald, Leann Silhan, Kevin R. Flaherty, Kevin Gibson, Mridu Gulati, Kalpalatha Guntupalli, Nishant Gupta, Amy Hajari Case, David Hotchkin, Tristan J. Huie, Robert J. Kaner, Hyun J. Kim, Lisa H. Lancaster, Mark Steele, Joseph A. Lasky, Doug Lee, Timothy Liesching, Randolph Lipchik, Jason Lobo, Tracy R. Luckhardt, Joao A. Andrade, Yolanda Mageto, Howard Huang, Prema Menon, Yolanda Mageto, Lake Morrison, Andrew Namen, Justin M. Oldham, Tessy Paul, David Zhang, Anna Podolanczuk, David Lederer, Nina M. Patel, Mary Porteous, Maryl Kreider, Rishi Raj, Paul Mohabir, Murali Ramaswamy, Tonya Russell, Paul Sachs, Zeenat Safdar, Shirin Shafazand, Marilyn Glassberg, Ather Siddiqi, Wael Asi, Barry Sigal, Mary E. Strek, Imre Noth, Sally Suliman, Jesse Roman, Jeremy Tabak, Rajat Walia, Timothy P. M. Whelan

**Affiliations:** 1 University of Minnesota, Minneapolis, MN USA; 2 Duke Clinical Research Institute, Durham, NC USA; 3 Duke University Medical Center, Durham, NC USA; 4 Section of Pulmonary and Critical Care Medicine, University of Chicago, Chicago, IL USA; 5 Boehringer Ingelheim Pharmaceuticals, Inc., Ridgefield, CT USA

**Keywords:** Interstitial lung disease, Mechanical ventilation, Mortality, Pulmonary fibrosis, Respiratory function tests

## Abstract

**Background:**

Hospitalizations are common among patients with idiopathic pulmonary fibrosis (IPF). We investigated the impact of hospitalizations on outcomes in patients with IPF.

**Methods:**

The IPF-PRO Registry is an observational US registry that enrolled patients with IPF that was diagnosed or confirmed at the enrolling center in the previous 6 months. Associations between patient characteristics and hospitalization, and between hospitalization and mortality, were analyzed using Cox regression models.

**Results:**

A total of 1002 patients with IPF were enrolled into the IPF-PRO Registry. Over a median follow-up time of 23.7 months (maximum: 67.0 months), 568 patients (56.7%) had at least one hospitalization. Of these patients, 319 (56.2%) had at least one respiratory-related hospitalization and 120 (21.1%) had at least one hospitalization with ventilatory support. Younger age (HR 0.68 [95% CI 0.55, 0.84] per 5-year increase for patients < 62 years), lower BMI (0.96 [0.93, 0.98] per 1-point increase), lower FVC % predicted (0.90 [0.83, 0.97] per 10% increase), oxygen use at rest (2.85 [2.18, 3.72]) and history of pulmonary hypertension (2.02 [1.37, 2.96]) at enrollment were associated with an increased risk of respiratory-related hospitalization during follow-up. In a multivariable model, there was an eightfold increase in the risk of mortality during hospitalization or within 90 days of discharge compared with outside of this period. The risk of mortality associated with a respiratory hospitalization or a hospitalization with ventilatory support was even greater.

**Conclusions:**

Data from the IPF-PRO Registry demonstrate that hospitalizations are common among patients with IPF. The risk of mortality during hospitalization or within 90 days of discharge was high, particularly among patients who were hospitalized for a respiratory cause or received ventilatory support.

*Trial registration* ClinicalTrials.gov, NCT01915511. Registered 5 August 2013, https://clinicaltrials.gov/ct2/show/NCT01915511

**Supplementary Information:**

The online version contains supplementary material available at 10.1186/s12931-021-01851-4.

## Introduction

Idiopathic pulmonary fibrosis (IPF) is a chronic fibrosing interstitial lung disease associated with progressive decline in lung function and a poor prognosis [1]. Hospitalizations are common among patients with IPF and are associated with high mortality, particularly in patients who require intensive care or mechanical ventilation [2–6].

The Idiopathic Pulmonary Fibrosis Prospective Outcomes (IPF-PRO) Registry (NCT01915511) is a multicenter, observational registry of patients with IPF in the US that aims to improve understanding of the clinical course and impact of IPF and current practices in its diagnosis and care [7]. Most previous studies assessing hospitalizations in patients with IPF have been based on databases of insurance claims or electronic medical records. Observational registries provide the opportunity to investigate the risk and impact of hospitalizations in clinical practice. We used data from the IPF-PRO Registry to evaluate the risk of hospitalization, the characteristics of patients who were hospitalized, the procedures and medications received during hospitalization, and post-hospitalization mortality in a large cohort of patients with IPF.

## Methods

The design of the IPF-PRO Registry has been published [7]. Briefly, 1002 patients with IPF that was diagnosed or confirmed at the enrolling center in the previous 6 months were enrolled between June 2014 and October 2018. Retrospective data were collected from patients’ medical records. Patients were then followed prospectively, with follow-up data collected approximately every 6 months until death, lung transplant, or withdrawal. Data for this analysis were extracted from the database in June 2020. The study was approved by the Duke University Institutional Review Board (Pro00046131). The protocol was approved by the relevant Institutional Review Boards and/or local Independent Ethics Committees prior to patient enrollment at each site listed in the Acknowledgments. All patients provided consent prior to entering the registry.

The Kaplan–Meier method was used to describe the frequency and timing of hospitalizations during the follow-up period. In descriptive analyses, we compared the demographic and clinical characteristics at enrollment into the registry of the patients who were and were not hospitalized during follow-up. Hospitalizations were categorized as having a respiratory or a non-respiratory cause (according to the investigator) and as with or without ventilatory support. Ventilatory support was designated if the investigator reported either “invasive ventilation” or “non-invasive ventilation”. Diagnostic tests, procedures and medications received during hospitalization, and discharge information for the first, second and third hospitalization, were assessed descriptively among all patients and in subgroups based on whether the hospitalization had a respiratory cause and whether the patient received ventilatory support.

Associations between patient characteristics at enrollment and the time to first hospitalization, first respiratory-related hospitalization, and first hospitalization with ventilatory support were analyzed using univariable, multivariable and parsimonious Cox regression models. The multivariable model included all the covariates. The parsimonious model included covariates identified following backward stepwise selection with an alpha-to-stay criterion of 0.05.

Associations between hospitalization (all, respiratory-related, with ventilatory support) and mortality during hospitalization or within 90, 180 and 360 days of discharge were analyzed using univariable and multivariable Cox regression models. In the analysis assessing the associations between hospitalizations and mortality, models were fit using the entire analysis cohort; in the analyses assessing the associations between respiratory-related hospitalizations and hospitalizations with ventilatory support, models were fit using the subset of patients with at least one hospitalization. The univariable model included hospitalization as a binary time-dependent covariate, the value of which switched from 0 to 1 at the time of hospitalization through the 90, 180, or 360 days following a hospitalization. Each threshold of time was examined in a separate model. All hospitalizations were considered while the patient remained in the risk set. The multivariable model included age, body mass index (BMI), forced vital capacity (FVC) % predicted, diffusing capacity of the lungs for carbon monoxide (DLco) % predicted, oxygen at rest, and history of coronary artery disease or heart failure at enrollment in addition to the time-dependent hospitalization covariate described above. These variables were identified through modeling of data from all patients in the IPF-PRO Registry (see Additional file 1: Appendix S1 for details).

In the Cox regression models, missing data were handled using multiple imputation. The multiple imputation was performed assuming that the data were missing at random with an arbitrary missing pattern. Using the Full Conditional Specification method, missing data were filled in five times to produce five data sets that were complete. Each complete data set was then analyzed using standard statistical analyses, the results of which were averaged to generate the final inferential results.

## Results

### Hospitalizations

A total of 1002 patients were enrolled into the IPF-PRO Registry at 46 sites. Data from one patient who was in hospital when enrolled, and who died during that hospitalization, were excluded from this analysis. The maximum follow-up time in the registry was 67.0 months and the median was 23.7 months. Over the follow-up period, 568 patients (56.7%) had at least one hospitalization (Additional file 1: Figure S1). Of these patients, 319 (56.2%) had at least one respiratory-related hospitalization (Additional file 1: Figure S2) and 120 (21.1%) had at least one hospitalization with ventilatory support (Additional file 1: Figure S3). Most patients who were hospitalized had one (54.0%) or two (21.3%) hospitalizations during follow-up. Among patients with at least one hospitalization, the first hospitalization had a respiratory cause in 250 patients (44.0%) and included provision of ventilatory support in 69 patients (12.1%). Of the 69 patients who received ventilatory support during their first hospitalization, 25 (36.2%) received invasive ventilation.

### Characteristics of hospitalized and non-hospitalized patients

Compared with those who were not hospitalized, a greater proportion of the patients who were hospitalized during follow-up were former smokers (68.8% vs 60.0%), used oxygen at rest (22.9% vs 15.9%) and used oxygen with activity (38.9% vs 28.4%) at enrollment (Table 1). Lung function at enrollment was similar in patients who were and were not hospitalized during follow-up (Table 1). Among those who were and were not hospitalized during follow-up, 27.1% and 19.4% of patients, respectively, had been hospitalized in the 12 months prior to enrollment.

**Table 1:** Demographic and clinical characteristics at enrollment of patients who were and were not hospitalized during follow-up in the IPF-PRO Registry

	Hospitalized during follow-up (n = 568)		Not hospitalized during follow-up (n = 433)	
	Measure	Missing data	Measure	Missing data
Male	419 (73.8)	0	328 (75.8)	0
Age, years	71 (66, 75)	0	70 (66, 76)	0
White	528 (93.0)	13 (2.3)	400 (92.4)	10 (2.3)
Body mass index, kg/m^2^	29.0 (25.9, 32.6)	21 (3.7)	28.8 (26.0, 31.8)	20 (4.6)
Smoking status		1 (0.2)		0
Current	10 (1.8)		8 (1.8)	
Former	391 (68.8)		260 (60.0)	
Never	166 (29.2)		165 (38.1)	
Private insurance	336 (59.2)	25 (4.4)	268 (61.9)	9 (2.1)
Diagnostic criteria for IPF^a^		2 (0.4)		2 (0.5)
Definite	376 (66.2)		278 (64.2)	
Possible/probable	190 (33.5)		153 (35.3)	
FVC % predicted	69.2 (58.5, 79.3)	35 (6.2)	71.4 (60.8, 84.7)	33 (7.6)
DLco % predicted	40.8 (30.9, 49.8)	58 (10.2)	44.5 (35.5, 54.3)	58 (13.4)
Oxygen use at rest	130 (22.9)	1 (0.2)	69 (15.9)	4 (0.9)
Oxygen use with activity	221 (38.9)	2 (0.4)	123 (28.4)	5 (1.2)
History of coronary artery disease or congestive heart failure	191 (33.6)	3 (0.5)	125 (28.9)	5 (1.2)
History of pulmonary hypertension	42 (7.4)	3 (0.5)	29 (6.7)	4 (0.9)
History of emphysema	72 (12.7)	5 (0.9)	54 (12.5)	4 (0.9)
History of sleep apnea	158 (27.8)	3 (0.5)	119 (27.5)	3 (0.7)

Data are n (%) or median (Q1, Q3)

^a^According to 2011 ATS/ERS/JRS/ALAT diagnostic guidelines [24]

### First hospitalizations during follow-up in the registry

The median (Q1, Q3) time from enrollment to the first hospital admission was 9.9 (4.2, 17.0) months. The median duration of the first hospitalization was 4 (2, 8) days. Among patients whose first hospitalization had a respiratory cause, the median (Q1, Q3) duration of hospitalization was 6 (3, 12) days. Among patients who received ventilatory support during their first hospitalization, the median (Q1, Q3) duration of hospitalization was 10 (6, 18) days. Median (Q1, Q3) duration of hospitalization was 18 (13, 28) days in patients who received invasive ventilation and 8 (3, 12) days in patients who received non-invasive ventilation.

Among patients whose first hospitalization had a respiratory cause, and who had data available on diagnostic tests and procedures, 35.6% had a chest CT, 28.0% had respiratory cultures performed, 25.6% had an echocardiogram, and 13.2% had a bronchoscopy during the hospitalization, while 10.0% and 17.6% of patients, respectively, received invasive and non-invasive ventilation (Table 2). Data on the outcome of hospitalization and on discharge destination were missing for 36.1% and 46.0% of patients, respectively. Among the 167 patients whose first hospitalization had a respiratory cause and who had data available on the outcome of hospitalization, 136 (81.4%) were discharged and, of those, 114 (83.8%) were discharged to home (Table 2).

**Table 2:** Characteristics of the first hospitalization

	Total (n = 568)		Respiratory-related hospitalization (n = 250)		Non-respiratory related hospitalization (n = 318)		Hospitalized with ventilatory support (n = 69)		Hospitalized without ventilatory support (n = 499)	
	Measure	Missing data	Measure	Missing data	Measure	Missing data	Measure	Missing data	Measure	Missing data
Hospitalized at enrolling center	208 (36.6)	21 (3.7)	122 (48.8)	1 (0.4)	86 (27.0)	20 (6.3)	43 (62.3)	0	165 (33.1)	21 (4.2)
Ventilator use		499 (87.9)		181 (72.4)		318 (100)		0		499 (100)
Invasive	25 (4.4)		25 (10.0)		0		25 (36.2)		0	
Non-invasive	44 (7.7)		44 (17.6)		0		44 (63.8)		0	
Diagnostic tests and procedures										
Chest CT	89 (15.7)	328 (57.7)	89 (35.6)	10 (4.0)	0	318 (100)	32 (46.4)	0	57 (11.4)	328 (65.7)
Bronchoscopy	33 (5.8)	329 (57.9)	33 (13.2)	11 (4.4)	0	318 (100)	22 (31.9)	0	11 (2.2)	329 (65.9)
Echocardiogram	64 (11.3)	329 (57.9)	64 (25.6)	11 (4.4)	0	318 (100)	25 (36.2)	0	39 (7.8)	329 (65.9)
Respiratory culture		498 (87.7)		180 (72.0)		318 (100)		34 (49.3)		464 (93.0)
Positive	18 (3.2)		18 (7.2)		0		9 (13.0)		9 (1.8)	
Negative	52 (9.2)		52 (20.8)		0		26 (37.7)		26 (5.2)	
Medications										
Antibiotics		327 (57.6)		9 (3.6)		318 (100)		0		327 (65.5)
Yes	149 (26.2)		149 (59.6)		0		47 (68.1)		102 (20.4)	
No	92 (16.2)		92 (36.8)		0		22 (31.9)		70 (14.0)	
Steroids		328 (57.7)		10 (4.0)		318 (100)		0		328 (65.7)
Yes	120 (21.1)		120 (48.0)		0		46 (66.7)		74 (14.8)	
No	120 (21.1)		120 (48.0)		0		23 (33.3)		97 (19.4)	
Anticoagulants		328 (57.7)		10 (4.0)		318 (100)		0		328 (65.7)
Yes	55 (9.7)		55 (22.0)		0		33 (47.8)		22 (4.4)	
No	185 (32.6)		185 (74.0)		0		36 (52.2)		149 (29.9)	
Outcome		205 (36.1)		83 (33.2)		122 (38.4)		10 (14.5)		195 (39.1)
Discharged	305 (53.7)		136 (54.4)		169 (53.1)		41 (59.4)		264 (52.9)	
Died	46 (8.1)		22 (8.8)		24 (7.5)		11 (15.9)		35 (7.0)	
Remained inpatient	12 (2.1)		9 (3.6)		3 (0.9)		7 (10.1)		5 (1.0)	
Discharge destination		261 (46.0)		112 (44.8)		149 (46.9)		28 (40.6)		233 (46.7)
Home	270 (47.5)		114 (45.6)		156 (49.1)		31 (44.9)		239 (47.9)	
Rehabilitation center	10 (1.8)		7 (2.8)		3 (0.9)		4 (5.8)		6 (1.2)	
Assisted living/nursing facility	5 (0.9)		2 (0.8)		3 (0.9)		1 (1.4)		4 (0.8)	
Another hospital	3 (0.5)		2 (0.8)		1 (0.3)		1 (1.4)		2 (0.4)	
Inpatient hospice	2 (0.4)		2 (0.8)		0		1 (1.4)		1 (0.2)	
Other	2 (0.4)		2 (0.8)		0		0		2 (0.4)	
Unknown	15 (2.6)		9 (3.6)		6 (1.9)		3 (4.3)		12 (2.4)	

Data are n (%)

### Second and third hospitalizations during follow-up in the registry

A total of 261 and 140 patients had a second and third hospitalization during the follow-up period, respectively. The median (Q1, Q3) times from enrollment to the second and third hospital admission were 15.5 (8.6, 25.1) and 19.3 (10.7, 28.1) months, respectively. The median duration of the second hospitalization was 4 days; the median duration of the third hospitalization was also 4 days. The diagnostic tests, procedures and medications received, and the proportions of patients discharged, for the second and third hospitalizations during the follow-up period are presented in Additional file 1: Tables S1 and S2.

### Association between patient characteristics and hospitalization

The results of the univariable and multivariable models are shown in Tables 3 and 4, and Additional file 1: Table S4. In the parsimonious models, not having private health insurance, lower DLco % predicted, and oxygen use at rest at enrollment, and hospitalization in the 12 months prior to enrollment, were associated with an increased risk of hospitalization during follow-up (Table 3). Younger age, lower BMI, lower FVC % predicted, oxygen use at rest and history of pulmonary hypertension at enrollment were associated with an increased risk of respiratory-related hospitalization during follow-up (Table 4). Younger age, lower FVC % predicted, lower DLco % predicted, and oxygen use at rest or with activity at enrollment were associated with an increased risk of hospitalization with ventilatory support (Additional file 1: Table S3).

**Table 3:** Association between patient characteristics at enrollment and hospitalization

	Univariable model		Multivariable model		Parsimonious model	
	HR (95% CI)	p-value	HR (95% CI)	p-value	HR (95% CI)	p-value
Female	1.02 (0.85, 1.24)	0.804	1.08 (0.89, 1.32)	0.424		
Age		0.017		0.217		
< 62 years, per 5-year increase	0.79 (0.65, 0.95)	0.014	0.84 (0.68, 1.02)	0.079		
≥ 62 years, per 5-year increase	1.09 (1.02, 1.17)	0.018	1.02 (0.95, 1.10)	0.568		
Hispanic/Latino ethnicity	1.23 (0.78, 1.92)	0.372	1.16 (0.72, 1.86)	0.541		
Body mass index, per 1-point increase	0.99 (0.97, 1.01)	0.287	0.99 (0.97, 1.01)	0.180		
Current/former smoker	1.27 (1.06, 1.52)	0.010	1.23 (1.02, 1.49)	0.033		
Private insurance	0.82 (0.69, 0.98)	0.029	0.84 (0.70, 1.02)	0.072	0.84 (0.70, 1.00)	0.046
Diagnostic criteria of definite IPF^a^	0.96 (0.80, 1.14)	0.630	0.96 (0.80, 1.14)	0.624		
FVC % predicted, per absolute 10% increase	0.89 (0.84, 0.94)	< 0.001	0.95 (0.89, 1.01)	0.101		
DLco % predicted, per absolute 10% increase	0.83 (0.77, 0.88)	< 0.001	0.92 (0.85, 1.00)	0.056	0.87 (0.81, 0.93)	< 0.001
Oxygen use at rest	1.99 (1.63, 2.44)	< 0.001	1.46 (1.12, 1.91)	0.005	1.63 (1.31, 2.03)	< 0.001
Oxygen use with activity	1.69 (1.42, 2.01)	< 0.001	1.21 (0.95, 1.53)	0.122		
History of coronary artery disease or congestive heart failure	1.19 (1.00, 1.42)	0.046	1.14 (0.95, 1.38)	0.161		
History of pulmonary hypertension	1.21 (0.88, 1.66)	0.239	0.87 (0.62, 1.22)	0.429		
History of emphysema	1.09 (0.85, 1.40)	0.476	0.95 (0.73, 1.23)	0.686		
History of sleep apnea	0.97 (0.81, 1.17)	0.751	0.96 (0.79, 1.18)	0.722		
Hospitalization in 12 months prior to enrollment	1.51 (1.25, 1.83)	< 0.001	1.30 (1.06, 1.58)	0.011	1.30 (1.08, 1.58)	0.007

Multivariable model included all the covariates listed. Parsimonious model included covariates selected after performing backwards selection on the multivariable model

^a^According to 2011 ATS/ERS/JRS/ALAT diagnostic guidelines [24]

**Table 4:** Association between patient characteristics at enrollment and respiratory-related hospitalization

	Univariable model		Multivariable model		Parsimonious model	
	HR (95% CI)	p-value	HR (95% CI)	p-value	HR (95% CI)	p-value
Female	1.12 (0.87, 1.44)	0.374	1.24 (0.95, 1.61)	0.124		
Age		< 0.001		< 0.001		< 0.001
< 62 years, per 5-year increase	0.62 (0.50, 0.77)	< 0.001	0.73 (0.58, 0.91)	0.005	0.68 (0.55, 0.84)	< 0.001
≥ 62 years, per 5-year increase	0.99 (0.90, 1.09)	0.810	0.91 (0.82, 1.01)	0.068	0.92 (0.83, 1.01)	0.085
Hispanic/Latino ethnicity	2.21 (1.34, 3.66)	0.002	1.43 (0.84, 2.45)	0.189		
Body mass index, per 1-point increase	0.98 (0.96, 1.00)	0.070	0.95 (0.93, 0.98)	< 0.001	0.96 (0.93, 0.98)	< 0.001
Current/former smoker	1.01 (0.79, 1.29)	0.927	1.04 (0.80, 1.36)	0.763		
Private insurance	1.00 (0.79, 1.27)	0.987	0.95 (0.72, 1.24)	0.689		
Diagnostic criteria of definite IPF^a^	1.06 (0.83, 1.35)	0.645	1.10 (0.86, 1.42)	0.440		
FVC % predicted, per absolute 10% increase	0.82 (0.76, 0.89)	< 0.001	0.93 (0.85, 1.01)	0.080	0.90 (0.83, 0.97)	0.005
DLco % predicted, per absolute 10% increase	0.75 (0.69, 0.83)	< 0.001	0.90 (0.80, 1.00)	0.057		
Oxygen use at rest	2.88 (2.24, 3.70)	< 0.001	2.32 (1.65, 3.25)	< 0.001	2.85 (2.18, 3.72)	< 0.001
Oxygen use with activity	2.31 (1.84, 2.91)	< 0.001	1.32 (0.96, 1.80)	0.087		
History of coronary artery disease or congestive heart failure	0.83 (0.66, 1.06)	0.133	0.87 (0.67, 1.13)	0.300		
History of pulmonary hypertension	2.37 (1.63, 3.43)	< 0.001	1.69 (1.10, 2.60)	0.016	2.02 (1.37, 2.96)	< 0.001
History of emphysema	1.11 (0.80, 1.55)	0.530	0.79 (0.54, 1.15)	0.216		
History of sleep apnea	1.00 (0.78, 1.28)	0.989	1.19 (0.90, 1.57)	0.221		
Hospitalization in 12 months prior to enrollment	1.17 (0.91, 1.50)	0.221	0.91 (0.69, 1.20)	0.501		

Multivariable model included all the covariates listed. Parsimonious model included covariates selected after performing backwards selection on the multivariable model

^a^According to 2011 ATS/ERS/JRS/ALAT diagnostic guidelines [24]

### Mortality

The Kaplan–Meier estimated rates of death at month 60 were 30.8% among patients with at least one hospitalization and 18.0% among non-hospitalized patients.

**Fig. 1:**
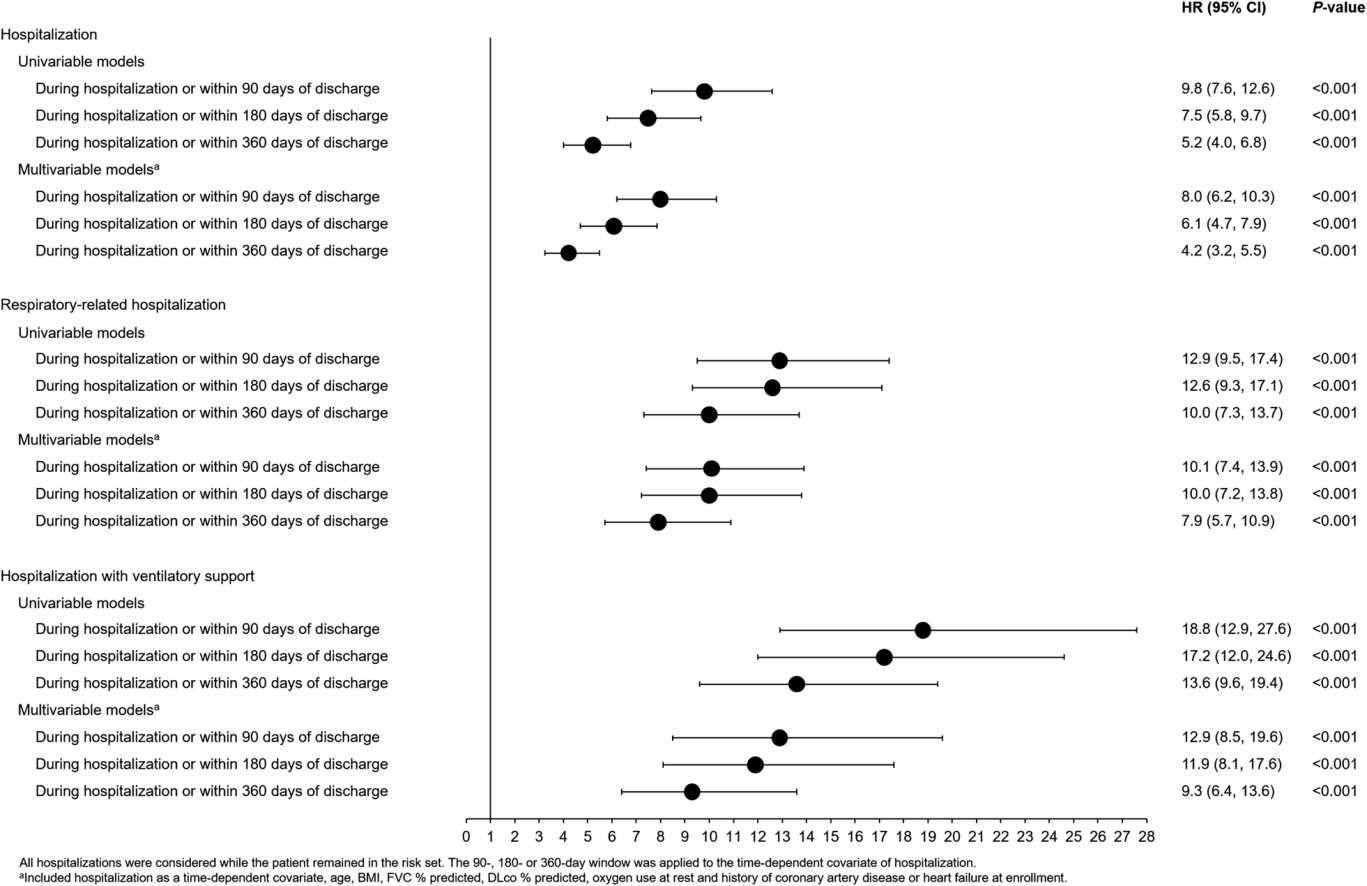
Associations between hospitalization and mortality

In both univariable and multivariable models, there were significant associations between hospitalization, respiratory-related hospitalization and hospitalization with ventilatory support and risk of mortality during the hospitalization or within 90, 180 and 360 days of discharge (Fig. 1). In multivariable models, there was an eightfold increase in the risk of mortality during hospitalization or within 90 days of discharge compared with the risk of mortality outside this period. There was a tenfold increase in the risk of mortality during a respiratory-related hospitalization or within 90 days of discharge compared with the risk of mortality outside this period among patients with at least one hospitalization. There was a 13-fold increase in the risk of mortality during hospitalization with ventilatory support or within 90 days of discharge compared with the risk of mortality outside this period among patients with at least one hospitalization.

## Discussion

We used data from the IPF-PRO Registry to evaluate the risk and impact of hospitalizations in patients with IPF. To our knowledge, the data from the IPF-PRO Registry are the first to assess associations between patient characteristics and risk of hospitalization, and outcomes following hospitalization, among patients with IPF in a registry setting. Hospitalizations were common in patients enrolled in this registry, with 24% of patients hospitalized in the year prior to enrollment, and 57% of patients hospitalized at least once over a median follow-up of 23.7 months. These data are consistent with analyses of insurance claims databases, which have reported rates of hospitalization among patients with IPF ranging from 38 to 49% over a 1-year period [8–10].

In our analyses, younger age, lower BMI, lower FVC, oxygen use at rest, and a history of pulmonary hypertension at enrollment were associated with an increased risk of respiratory-related hospitalization. As would be expected, more severe lung function impairment at enrollment, as shown by lower FVC, lower DLco, or oxygen use, was associated with a greater risk of hospitalization with ventilatory support. Younger age was also associated with a greater risk of hospitalization with ventilatory support. This is consistent with data from the US Nationwide Inpatient Sample, which demonstrated a greater likelihood of mechanical ventilation in patients with IPF who were younger [3]. Our observation of a greater risk of hospitalization in the youngest patients is consistent with a previous analysis of data from the IPF-PRO Registry in which there was an increase in the risk of mortality in patients aged under 60 years [11]. We may speculate that the youngest patients represent a different cohort to the elderly patients, with disease that is more often familial than a disease of aging and that is associated with worse outcomes.

In the IPF-PRO Registry, the median length of stay in hospital was 4 days. Median length of stay was longer among patients who were hospitalized for a respiratory cause or who received ventilatory support (6 and 10 days, respectively). These findings are similar to the length of hospital stay reported in other observational studies in patients with IPF [4, 5, 12–14]. Analyses of the Premier Healthcare Database and the Nationwide Inpatient Sample, two broadly representative datasets of hospitalized patients in the US, found that the median/mean length of hospital stay among patients with IPF was 5 and 7.4 days, respectively [5, 14].

The most common procedures undertaken during respiratory-related hospitalizations in the IPF-PRO Registry were chest CT, respiratory cultures and echocardiogram. While it may seem surprising that almost two-thirds of patients with IPF who were hospitalized for a respiratory cause and had available data on diagnostic tests and procedures did not receive a CT, similarly low rates of chest CT in hospitalized patients with IPF have been observed in other studies [5, 15]. This may indicate that many clinicians do not request a CT scan if they do not think the results are necessary to inform patient management.

Among patients with data available, 54% of the hospitalized patients in our study were discharged, with most discharged to their home. Data from the US Nationwide Inpatient Sample found that 46% of patients with IPF who were hospitalized were routinely discharged and that 18% required home healthcare after discharge [14]. Our analyses showed a high risk of mortality in the year following hospital discharge, particularly among patients who were hospitalized for a respiratory cause or who received ventilatory support. Patients who were hospitalized had an eightfold increase in the risk of mortality during hospitalization or within 90 days of discharge compared with patients who were not hospitalized. Other studies have also found high post-hospitalization mortality in patients with IPF. Data from a single-center study showed that among 134 patients with fibrotic interstitial lung diseases including IPF who were hospitalized following acute respiratory worsening and survived to discharge, median time to death post-discharge was 9.4 months [16]. An analysis of data from 150 patients with IPF hospitalized at a tertiary referral center demonstrated much shorter survival among patients who survived a respiratory than a non-respiratory admission (median 9.1 versus 43.5 months) [12]. In-hospital mortality rates among patients with IPF have been reported to be approximately 13% to 15% [4, 5, 13–15, 17] and to be particularly high in patients who receive mechanical ventilation [3, 6, 12, 18]. Based on data from the Premier Healthcare Database, mechanical ventilation was associated with, on average, a more than fivefold increase in the risk of in-hospital mortality [4, 5].

Our analyses have several limitations. Due to the challenges inherent in real-world studies, there were substantial missing data on in-hospital procedures, medications and discharges. The definition of a respiratory versus non-respiratory related hospitalization was based solely on investigator report. We are unable to determine which hospitalizations were due to acute exacerbations of IPF or respiratory infections. Associations between patient characteristics and risk of hospitalization were based on data collected at enrollment and the impact of changes in functional parameters were not assessed. The impact of antifibrotic therapy, which has been shown to reduce the risk of acute exacerbations and respiratory-related hospitalizations [19–21] and mortality [21–23], on mortality during and following hospitalization was not assessed.

## Conclusions

Data from the IPF-PRO Registry demonstrate that hospitalizations are common among patients with IPF. Younger age, lower BMI, lower FVC, oxygen use at rest, and a history of pulmonary hypertension at enrollment were associated with an increased risk of respiratory hospitalization. The risk of mortality during hospitalization or within 90 days following discharge was high, particularly among patients who were hospitalized for a respiratory cause or who received ventilatory support. A podcast of Dr. Hyun Kim discussing these data is available at: https://www.usscicomms.com/respiratory/kim/IPF-PROhospitalizations.

## Supplementary Information

**Additional file 1. Appendix S1**. Identification of variables for inclusion in the multivariable Cox regression model assessing associations between hospitalization and mortality.** Table S1**. Characteristics of the second hospitalization.** Table S2**. Characteristics of the third hospitalization.** Table S3**. Association between patient characteristics at enrollment and hospitalization with ventilatory support.** Figure S1**. Time to first hospitalization.** Figure S2**. Time to first respiratory-related hospitalization.** Figure S3**. Time to first hospitalization with ventilatory support.

## Data Availability

The datasets analyzed during the current study are not publicly available, but are available from the corresponding author on reasonable request.

## Abbreviations

BMI: Body mass index
DLco: Diffusing capacity of the lungs for carbon monoxide
FVC: Forced vital capacity
HR: Hazard ratio
IPF: Idiopathic pulmonary fibrosis
