# Supplementary material for: Hospitalizations in patients with idiopathic pulmonary fibrosis
Source: Respir Res. 2021 Sep 30;22:257. doi: 10.1186/s12931-021-01851-4 (PMC8481759; doi:10.1186/s12931-021-01851-4)
Supplement: Supplementary file 1 — Additional file 1. Appendix S1. Identification of variables for inclusion in the multivariable Cox regression model assessing associations between hospitalization and mortality. Table S1. Characteristics of the second hospitalization. Table S2. Characteristics of the third hospitalization. Table S3. Association between patient characteristics at enrollment and hospitalization with ventilatory support. Figure S1. Time to first hospitalization. Figure S2. Time to first respiratory-related hospitalization. Figure S3. Time to first hospitalization with ventilatory support. [file 12931_2021_1851_MOESM1_ESM.docx]

**Additional file 1**

**Hospitalizations in patients with idiopathic pulmonary fibrosis**

Hyun J Kim, Laurie D Snyder, Ayodeji Adegunsoye, Megan L Neely, Shaun Bender, Eric S White, Craig S Conoscenti, Mary E Strek, on behalf of the IPF-PRO Registry investigators

**Appendix S1:** Identification of variables for inclusion in the multivariable Cox regression model assessing associations between hospitalization and mortality

Candidate predictors for the multivariable model were identified based on clinical expertise, review of the literature, and the number of events in the IPF-PRO Registry cohort (*i.e.,* at least 5 events per predictor were required). An initial set of 21 candidate predictors, all measured at the time of enrollment, was identified, comprising demographics (age, sex, insurance type, distance to enrolling center); IPF history and severity (new diagnosis of IPF at enrolling center versus confirmed diagnosis of IPF at enrolling center, diagnostic criteria for IPF [definite versus probable or possible according to 2011 ATS/ERS/JRS/ALAT guidelines], time from symptom onset to new or confirmed diagnosis of IPF at enrolling center, forced expiratory volume in 1 second [FEV_1_] % predicted, forced vital capacity [FVC] % predicted, diffusing capacity of the lungs for carbon monoxide [DLco] % predicted, supplemental oxygen use with activity, supplemental oxygen use at rest); comorbidities and other risk factors (body mass index, cardiac disease, pulmonary hypertension, clinically significant emphysema on high-resolution computed tomography (HRCT) in the opinion of the investigator, pulmonary embolism or deep vein thrombosis, gastro-esophageal reflux disease, smoking history); and hospitalization history (any hospitalization in the 12 months before enrollment, number of respiratory hospitalizations in the 12 months before enrollment). All covariates were entered into the Cox proportional hazards model and backwards selection then applied using an alpha-to-stay criterion of 0.05.

**Table S1.** Characteristics of the second hospitalization

|  | **Total**  **(n=261)** | | **Respiratory-related hospitalization  (n=107)** | | **Non-respiratory related hospitalization (n=154)** | | **Hospitalized with ventilatory support  (n=27)** | | **Hospitalized without ventilatory support (n=234)** | |
| --- | --- | --- | --- | --- | --- | --- | --- | --- | --- | --- |
|  | **Measure** | **Missing data** | **Measure** | **Missing data** | **Measure** | **Missing data** | **Measure** | **Missing data** | **Measure** | **Missing data** |
| Hospitalized at enrolling center | 97 (37.2) | 0 | 54 (50.5) | 0 | 43 (27.9) | 0 | 20 (74.1) | 0 | 77 (32.9) | 0 |
| Ventilator use |  | 234 (89.7) |  | 81 (75.7) |  | 153 (99.4) |  | 0 |  | 234 (100) |
| Invasive | 11 (4.2) |  | 10 (9.3) |  | 1 (0.6) |  | 11 (40.7) |  | 0 |  |
| Non-invasive | 16 (6.1) |  | 16 (15.0) |  | 0 |  | 16 (59.3) |  | 0 |  |
| Diagnostic tests and procedures |  |  |  |  |  |  |  |  |  |  |
| Chest HRCT | 48 (18.4) | 152 (58.2) | 46 (43.0) | 1 (0.9) | 2 (1.3) | 151 (98.1) | 12 (44.4) | 0 | 36 (15.4) | 152 (65.0) |
| Bronchoscopy | 8 (3.1) | 154 (59.0) | 8 (7.5) | 3 (2.8) | 0 | 151 (98.1) | 6 (22.2) | 0 | 2 (0.9) | 154 (65.8) |
| Echocardiogram | 27 (10.3) | 152 (58.2) | 26 (24.3) | 1 (0.9) | 1 (0.6) | 151 (98.1) | 7 (25.9) | 0 | 20 (8.5) | 152 (65.0) |
| Respiratory culture |  | 233 (89.3) |  | 80 (74.8) |  | 153 (99.4) |  | 17 (63.0) |  | 216 (92.3) |
| Positive | 6 (2.3) |  | 6 (5.6) |  | 0 |  | 1 (3.7) |  | 5 (2.1) |  |
| Negative | 22 (8.4) |  | 21 (19.6) |  | 1 (0.6) |  | 9 (33.3) |  | 13 (5.6) |  |
| Medications |  |  |  |  |  |  |  |  |  |  |
| Antibiotics |  | 153 (58.6) |  | 2 (1.9) |  | 151 (98.1) |  | 0 |  | 153 (65.4) |
| Yes | 67 (25.7) |  | 67 (62.6) |  | 0 |  | 19 (70.4) |  | 48 (20.5) |  |
| No | 41 (15.7) |  | 38 (35.5) |  | 3 (1.9) |  | 8 (29.6) |  | 33 (14.1) |  |
| Steroids |  | 153 (58.6) |  | 2 (1.9) |  | 151 (98.1) |  | 0 |  | 153 (65.4) |
| Yes | 60 (23.0) |  | 58 (54.2) |  | 2 (1.3) |  | 19 (70.4) |  | 41 (17.5) |  |
| No | 48 (18.4) |  | 47 (43.9) |  | 1 (0.6) |  | 8 (29.6) |  | 40 (17.1) |  |
| Anticoagulants |  | 153 (58.6) |  | 2 (1.9) |  | 151 (98.1) |  | 0 |  | 153 (65.4) |
| Yes | 20 (7.7) |  | 20 (18.7) |  | 0 |  | 11 (40.7) |  | 9 (3.8) |  |
| No | 88 (33.7) |  | 85 (79.4) |  | 3 (1.9) |  | 16 (59.3) |  | 72 (30.8) |  |
| Outcome |  | 84 (32.2) |  | 29 (27.1) |  | 55 (35.7) |  | 4 (14.8) |  | 80 (34.2) |
| Discharged | 157 (60.2) |  | 62 (57.9) |  | 95 (61.7) |  | 18 (66.7) |  | 139 (59.4) |  |
| Died | 15 (5.7) |  | 12 (11.2) |  | 3 (1.9) |  | 4 (14.8) |  | 11 (4.7) |  |
| Remained inpatient | 5 (1.9) |  | 4 (3.7) |  | 1 (0.6) |  | 1 (3.7) |  | 4 (1.7) |  |
| Discharge destination |  | 102 (39.1) |  | 44 (41.1) |  | 58 (37.7) |  | 8 (29.6) |  | 94 (40.2) |
| Home | 140 (53.6) |  | 53 (49.5) |  | 87 (56.5) |  | 14 (51.9) |  | 126 (53.8) |  |
| Rehabilitation center | 4 (1.5) |  | 2 (1.9) |  | 2 (1.3) |  | 2 (7.4) |  | 2 (0.9) |  |
| Assisted living/ nursing facility | 4 (1.5) |  | 0 |  | 4 (2.6) |  | 0 |  | 4 (1.7) |  |
| Another hospital | 0 |  | 0 |  | 0 |  | 0 |  | 0 |  |
| Inpatient hospice | 2 (0.8) |  | 2 (1.9) |  | 0 |  | 1 (3.7) |  | 1 (0.4) |  |
| Other | 5 (1.9) |  | 4 (3.7) |  | 1 (0.6) |  | 1 (3.7) |  | 4 (1.7) |  |
| Unknown | 4 (1.5) |  | 2 (1.9) |  | 2 (1.3) |  | 1 (3.7) |  | 3 (1.3) |  |

Data are n (%).

**Table S2.** Characteristics of the third hospitalization

|  | **Total**  **(n=140)** | | **Respiratory-related hospitalization  (n=66)** | | **Non-respiratory related hospitalization (n=74)** | | **Hospitalized with ventilatory support  (n=21)** | | **Hospitalized without ventilatory support (n=119)** | |
| --- | --- | --- | --- | --- | --- | --- | --- | --- | --- | --- |
|  | **Measure** | **Missing data** | **Measure** | **Missing data** | **Measure** | **Missing data** | **Measure** | **Missing data** | **Measure** | **Missing data** |
| Hospitalized at enrolling center | 62 (44.3) | 1 (0.7) | 36 (54.5) | 0 | 26 (35.1) | 1 (1.4) | 13 (61.9) | 0 | 49 (41.2) | 1 (0.8) |
| Ventilator use |  | 119 (85.0) |  | 45 (68.2) |  | 74 (100.0) |  | 0 |  | 119 (100) |
| Invasive | 7 (5.0) |  | 7 (10.6) |  | 0 |  | 7 (33.3) |  | 0 |  |
| Non-invasive | 14 (10.0) |  | 14 (21.2) |  | 0 |  | 14 (66.7) |  | 0 |  |
| Diagnostic tests and procedures |  |  |  |  |  |  |  |  |  |  |
| Chest HRCT | 28 (20.0) | 75 (53.6) | 28 (42.4) | 2 (3.0) | 0 | 73 (98.6) | 12 (57.1) | 0 | 16 (13.4) | 75 (63.0) |
| Bronchoscopy | 6 (4.3) | 76 (54.3) | 6 (9.1) | 3 (4.5) | 0 | 73 (98.6) | 5 (23.8) | 1 (4.8) | 1 (0.8) | 75 (63.0) |
| Echocardiogram | 15 (10.7) | 76 (54.3) | 14 (21.2) | 3 (4.5) | 1 (1.4) | 73 (98.6) | 8 (38.1) | 1 (4.8) | 7 (5.9) | 75 (63.0) |
| Respiratory culture |  | 118 (84.3) |  | 44 (66.7) |  | 74 (100.0) |  | 9 (42.9) |  | 109 (91.6) |
| Positive | 6 (4.3) |  | 6 (9.1) |  | 0 |  | 3 (14.3) |  | 3 (2.5) |  |
| Negative | 16 (11.4) |  | 16 (24.2) |  | 0 |  | 9 (42.9) |  | 7 (5.9) |  |
| Medications |  |  |  |  |  |  |  |  |  |  |
| Antibiotics |  | 75 (53.6) |  | 2 (3.0) |  | 73 (98.6) |  | 0 |  | 75 (63.0) |
| Yes | 38 (27.1) |  | 38 (57.6) |  | 0 |  | 16 (76.2) |  | 22 (18.5) |  |
| No | 27 (19.3) |  | 26 (39.4) |  | 1 (1.4) |  |  |  |  |  |
| Steroids |  | 76 (54.3) |  | 3 (4.5) |  | 73 (98.6) |  | 1 (4.8) |  | 75 (63.0) |
| Yes | 39 (27.9) |  | 39 (59.1) |  | 0 |  | 15 (71.4) |  | 24 (20.2) |  |
| No | 25 (17.9) |  | 24 (36.4) |  | 1 (1.4) |  |  |  |  |  |
| Anticoagulants |  | 76 (54.3) |  | 3 (4.5) |  | 73 (98.6) |  | 1 (4.8) |  | 75 (63.0) |
| Yes | 16 (11.4) |  | 16 (24.2) |  | 0 |  | 10 (47.6) |  | 6 (5.0) |  |
| No | 48 (34.3) |  | 47 (71.2) |  | 1 (1.4) |  |  |  |  |  |
| Outcome |  | 48 (34.3) |  | 19 (28.8) |  | 29 (39.2) |  | 3 (14.3) |  | 45 (37.8) |
| Discharged | 82 (58.6) |  | 39 (59.1) |  | 43 (58.1) |  | 15 (71.4) |  | 67 (56.3) |  |
| Died | 9 (6.4) |  | 7 (10.6) |  | 2 (2.7) |  | 3 (14.3) |  | 6 (5.0) |  |
| Remained inpatient | 1 (0.7) |  | 1 (1.5) |  | 0 |  | 0 |  | 1 (0.8) |  |
| Discharge destination |  | 57 (40.7) |  | 27 (40.9) |  | 30 (40.5) |  | 6 (28.6) |  | 51 (42.9) |
| Home | 75 (53.6) |  | 37 (56.1) |  | 38 (51.4) |  | 14 (66.7) |  | 61 (51.3) |  |
| Rehabilitation center | 3 (2.1) |  | 1 (1.5) |  | 2 (2.7) |  | 0 |  | 3 (2.5) |  |
| Assisted living/ nursing facility | 1 (0.7) |  | 0 |  | 1 (1.4) |  | 0 |  | 1 (0.8) |  |
| Another hospital | 1 (0.7) |  | 0 |  | 1 (1.4) |  | 0 |  | 1 (0.8) |  |
| Inpatient hospice | 0 |  | 0 |  | 0 |  | 0 |  | 0 |  |
| Other | 2 (1.4) |  | 1 (1.5) |  | 1 (1.4) |  | 1 (4.8) |  | 1 (0.8) |  |
| Unknown | 1 (0.7) |  | 0 |  | 1 (1.4) |  | 0 |  | 1 (0.8) |  |

Data are n (%).

**Table S3.** Association between patient characteristics at enrollment and hospitalization with ventilatory support

|  | **Univariable model** | | **Multivariable model** | | **Parsimonious model** | |
| --- | --- | --- | --- | --- | --- | --- |
|  | **HR (95% CI)** | **P-value** | **HR (95% CI)** | **P-value** | **HR (95% CI)** | **P-value** |
| Female sex | 1.12 (0.75, 1.66) | 0.591 | 1.24 (0.81, 1.89) | 0.324 |  |  |
| Age |  | <0.001 |  | <0.001 |  | <0.001 |
| <62 years, per 5-year increase | 0.60 (0.46, 0.78) | <0.001 | 0.73 (0.55, 0.97) | 0.029 | 0.76 (0.58, 1.00) | 0.054 |
| ≥62 years, per 5-year increase | 0.77 (0.64, 0.91) | 0.003 | 0.73 (0.60, 0.88) | 0.001 | 0.73 (0.61, 0.88) | <0.001 |
| Hispanic/Latino ethnicity | 3.09 (1.57, 6.08) | 0.001 | 1.67 (0.78, 3.58) | 0.184 |  |  |
| Body mass index, per 1-point increase | 1.02 (0.99, 1.06) | 0.190 | 1.00 (0.96, 1.04) | 0.946 |  |  |
| Current/former smoker | 0.97 (0.65, 1.44) | 0.882 | 0.85 (0.55, 1.32) | 0.476 |  |  |
| Private insurance | 1.04 (0.71, 1.54) | 0.827 | 0.78 (0.51, 1.18) | 0.239 |  |  |
| Diagnostic criteria of definite IPF* | 1.24 (0.82, 1.86) | 0.309 | 1.50 (0.97, 2.30) | 0.067 |  |  |
| FVC % predicted, per 10% absolute increase | 0.66 (0.58, 0.76) | <0.001 | 0.81 (0.69, 0.95) | 0.009 | 0.84 (0.73, 0.97) | 0.019 |
| DLco % predicted, per 10% absolute increase | 0.64 (0.55, 0.75) | <0.001 | 0.81 (0.66, 0.99) | 0.042 | 0.78 (0.65, 0.95) | 0.012 |
| Oxygen use at rest | 3.32 (2.26, 4.88) | <0.001 | 1.60 (0.97, 2.65) | 0.065 | 1.69 (1.05, 2.73) | 0.031 |
| Oxygen use with activity | 3.55 (2.44, 5.17) | <0.001 | 1.84 (1.13, 3.01) | 0.015 | 1.90 (1.18, 3.06) | 0.009 |
| History of coronary artery disease or congestive heart failure | 0.73 (0.49, 1.09) | 0.127 | 0.97 (0.61, 1.53) | 0.893 |  |  |
| History of pulmonary hypertension | 1.95 (1.04, 3.64) | 0.037 | 1.42 (0.69, 2.95) | 0.343 |  |  |
| History of emphysema | 1.56 (0.95, 2.56) | 0.078 | 1.31 (0.74, 2.33) | 0.354 |  |  |
| History of sleep apnea | 0.82 (0.54, 1.24) | 0.346 | 0.83 (0.51, 1.35) | 0.452 |  |  |
| Hospitalization in 12 months prior to enrollment | 1.16 (0.77, 1.76) | 0.466 | 0.78 (0.49, 1.25) | 0.306 |  |  |

Multivariable model included all the covariates listed. Parsimonious model included covariates selected after performing backwards selection on the multivariable model.

*According to 2011 ATS/ERS/JRS/ALAT diagnostic guidelines [1].

**Figure S1.** Time to first hospitalization


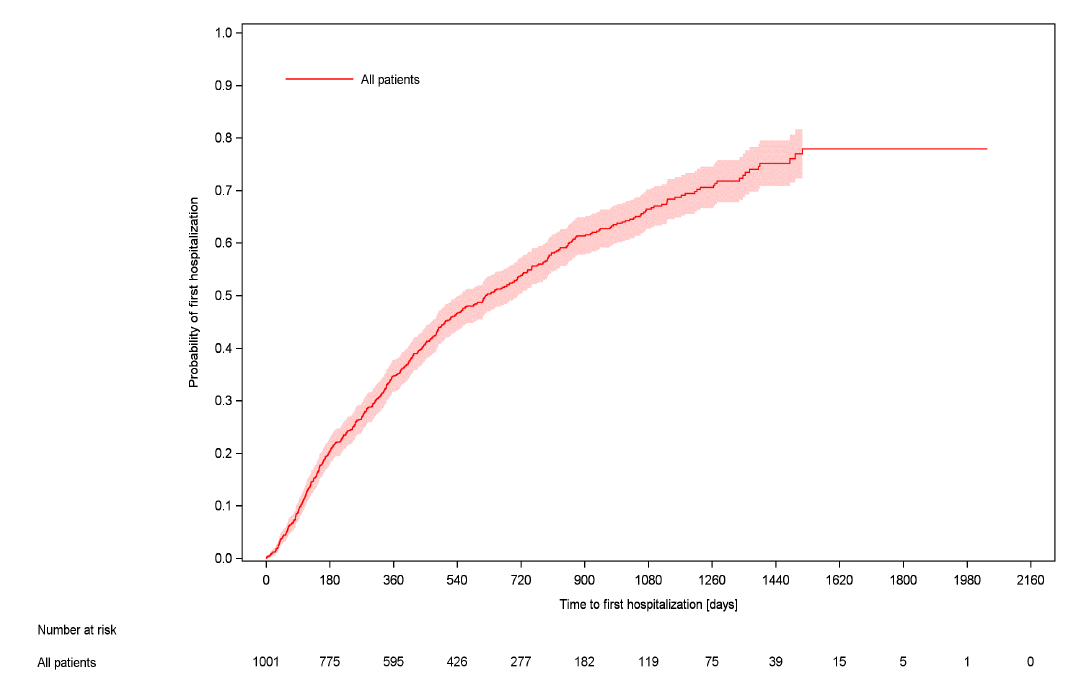


**Figure S2.** Time to first respiratory-related hospitalization


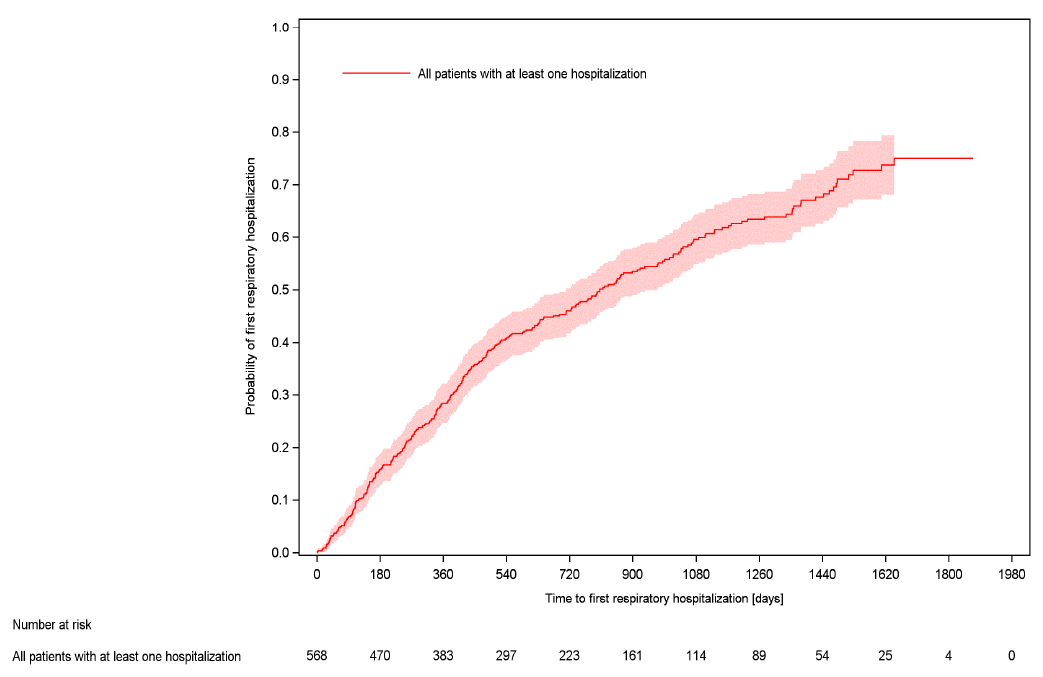


**Figure S3.** Time to first hospitalization with ventilatory support


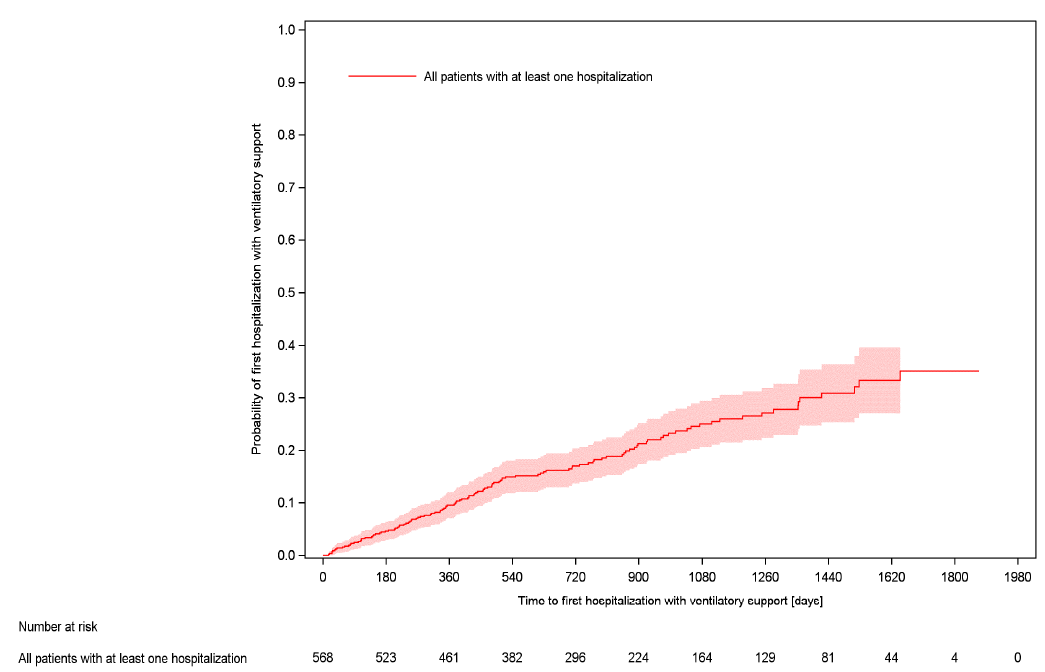


**References**

1. Raghu G, Collard HR, Egan JJ, et al. An official ATS/ERS/JRS/ALAT statement: idiopathic pulmonary fibrosis: evidence-based guidelines for diagnosis and management. Am J Respir Crit Care Med 2011;183(6):788–824.
